# Histamine-induced RPS6 phosphorylation in dendritic cells is associated with the severity of peanut allergic reactions

**DOI:** 10.1172/jci.insight.196167

**Published:** 2025-09-11

**Authors:** Florent Fauchère, Andreas Thiel, Margitta Worm, Julian Braun

**Affiliations:** 1 Der Simulierte Mensch, a science framework of Technische Universität Berlin and Charité – Universitätsmedizin Berlin, Berlin, Germany.; 2 Regenerative Immunology and Aging, Berlin Institute of Health Center for Immunomics, and; 3 Division of Allergy and Immunology, Department of Dermatology, Venerology and Allergy, Charité – Universitätsmedizin Berlin, corporate member of Freie Universität Berlin and Humboldt-Universität zu Berlin, Berlin, Germany.; 4 Project KFO 339: Food Allergy and Tolerance (Food@) (detailed in Supplemental Acknowledgments).

**Keywords:** Cell biology, Immunology, Allergy, Basophils, Dendritic cells

## Abstract

The phosphorylation of the ribosomal protein S6 (RPS6) was reported to be increased in myeloid cell subsets after stimulation with peanut extract in peanut-allergic individuals or with anti-IgE antibodies in both allergic and nonallergic donors. The mechanisms driving this increase of RPS6 phosphorylation (pS6) and its clinical impacts remain to be elucidated. Therefore, we investigated the mechanism of pS6 induction in plasmacytoid DCs (pDCs) and conventional DCs (cDCs) using whole blood stimulated with peanut extract or anti-IgE antibodies. This approach included in vitro basophil depletion and the application of receptor antagonists. Clinical associations with differential pS6 were performed with data from a well-defined cohort of peanut-allergic individuals participating in the food intervention trial TINA. Our findings revealed an increase of pS6 in pDCs and cDCs via histamine receptor 2 (H2R) signaling after IgE-dependent basophil degranulation and histamine release. In adults — but not in children — pS6 in cDCs was positively associated with food allergy severity, as determined by titrated oral food challenges. The association of pS6 in cDCs with food allergy severity in an age-dependent manner suggests a possibly novel functional mechanism, which may contribute to the course of food allergy, e.g., via increased antigen presentation.

## Abstract 2

<span>RPS6 phosphorylation increases in dendritic cells via histamine signaling after IgE-mediated basophil activation, and correlates in cDCs with food allergy severity in adult allergic individuals</span>

## Graphical abstract

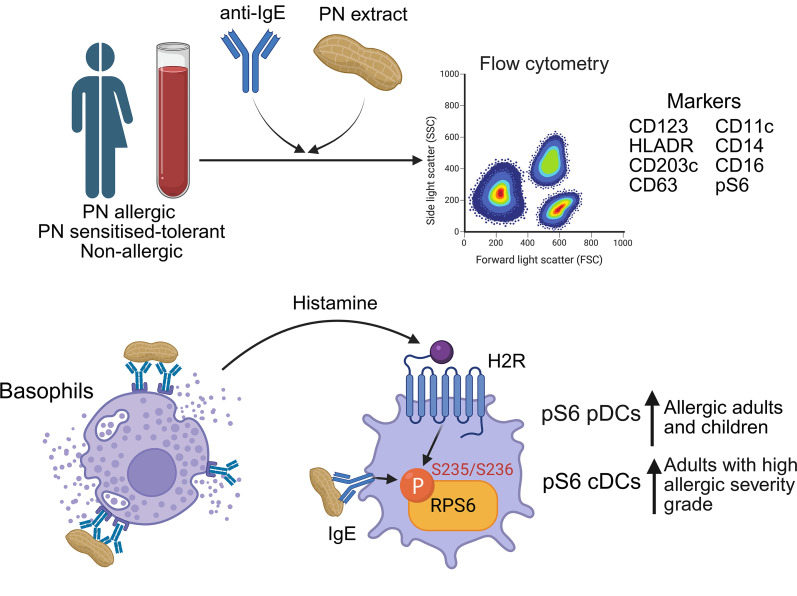

## Introduction

The incidence of IgE-mediated food allergy is rising in industrialized countries (1, 2) leading to life-threatening anaphylactic reactions driven by the cross-linking of the high-affinity IgE receptor (FCεR1) on mast cells and basophils. As mast cells are not present in sufficient numbers in the peripheral blood, basophils are often used to study effector functions in allergy. In particular, the basophil activation test (BAT) is an established diagnostic assay measuring basophil degranulation in response to increasing allergen concentrations (3). Upon cross-linking of FCεR1α, basophils become activated and release preformed mediators, such as histamine. Moreover, they are capable of the secretion of various early mediators, including leukotriene C4 (LTC4), platelet-activating factor (PAF), and, later, cytokines like IL-3 or IL-4 (4, 5). In that manner, basophils contribute to both the early and late phases of allergic reactions, modulating the immune response.

Recently, an increase of phosphorylation of the 40S ribosomal protein S6 (RPS6) in basophils but also myeloid cell subsets, such as plasmacytoid DCs (pDCs), conventional DCs (cDCs), and monocytes, following the stimulation of whole blood from allergic individuals with peanut extract (PN) or anti-IgE antibodies (aIgE) has been reported (6). These data suggested basophil degranulation as the primary cause of RPS6 phosphorylation (pS6), which controls several pathways of cellular homeostasis (7, 8). However, the cellular mechanisms of pS6 in myeloid cells in the context of food allergic reactions and its clinical relevance have not been identified yet.

In this study, we applied fluorescence-based flow cytometry in a well-characterized cohort of peanut-allergic individuals from the TINA intervention trial (9). By employing in vitro cellular depletion and receptor antagonists, we identified basophil-released histamine signaling through the histamine receptor 2 (H2R) as the primary mechanism of pS6 phosphorylation. As the increase of pS6 in cDCs was pronounced in peanut-allergic adults, but not children, with severe clinically proven reactions, our data suggest a possibly novel mechanism, which may contribute to the disease course of food allergy by enhancing antigen-presenting cell functions.

## Results

### Increased phosphorylation of RPS6 in pDCs and cDCs after allergenic stimulation.

**Figure 1:**
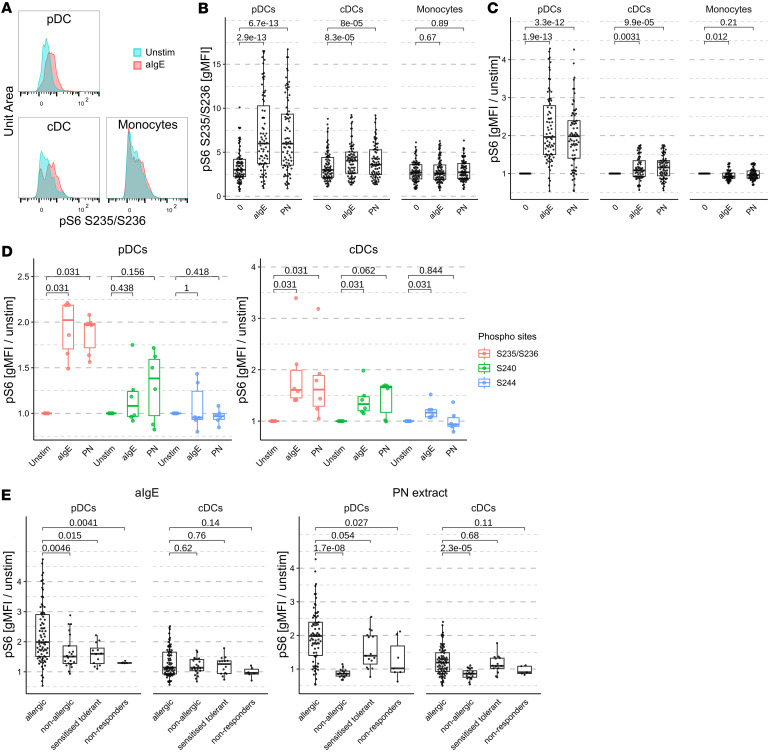
RPS6 in DCs of peanut-allergic donors is phosphorylated after stimulation of whole blood with anti-IgE antibodies or PN extract. (**A**) Histograms depicting RPS6 pS65/S236 staining in pDCs, cDCs, and classical monocytes (CD14^+^CD16^–^) after stimulation of blood with anti-IgE antibodies. (**B** and **C**) pS6 in peanut-allergic donor blood stimulated with anti-IgE antibodies (positive control) or PN extract, as (**B**) gMFI and (**C**) gMFI normalized to unstimulated (*n* = 73). *P* values are indicated. (**D**) Phosphorylation of RPS6 at the S235/S236, S240, or S244 sites in pDCs or cDCs (*n* = 6). Statistical tests were performed using paired Wilcoxon tests. *P* values are indicated. (**E**) Phosphorylation of RPS6 at the S235/S236 sites after stimulation of blood with anti-IgE antibodies or with PN extract, normalized to unstimulated blood, in PN-allergic (*n* = 73), nonallergic (*n* = 18), PN-sensitized but tolerant (*n* = 14), or PN-allergic basophils from nonresponder donors (*n* = 6). Statistical tests were performed using nonpaired Wilcoxon tests. *P* values are indicated.

To examine the immune response to PN, we designed a flow cytometry panel to conduct a modified BAT. This panel enabled the detection of pS6 at the S635/S236 sites and the identification of basophils (HLADR^–^, CD123^+^, CD203c^med/hi^, CD63^med/hi^), pDCs (CD123^+^, HLADR^+^, CD11c^–^, CD203c^–^), cDCs (HLADR^+^CD11c^+^CD14^–^CD16^–^, CD203c^–^) and classical, inflammatory, and intermediate monocytes (HLADR^+^, CD11c^+^, CD203c^–^, CD14^+/–^CD16^+/–^). Activated basophils were identified as CD63^hi^. The gating strategy and histograms representing pS6 mean fluorescence intensity in pDCs, cDCs, and monocytes are depicted in Figure 1A and Supplemental Figure 1A (supplemental material available online with this article; https://doi.org/10.1172/jci.insight.196167DS1), respectively. Unless otherwise specified, blood stimulation was conducted for 30 minutes.

In accordance with previous data, we observed an increase in pS6 at the S235/S236 site in pDCs and cDCs from peanut-allergic donors following stimulation with PN or anti-IgE antibodies (Figure 1B). In contrast, an induction of pS6 in monocytes was detected only in some donors. To address the high variability of pS6 at baseline, we normalized the pS6 geometric mean fluorescence intensity (gMFI) to the unstimulated sample, expressed as fold change (Figure 1C). While most donors showed a strongly increased pS6 in pDCs (median fold change ~2), the effect on cDCs was more modest and observed in fewer than three-quarters of the donors (median fold change ~1.2).

RPS6 can be phosphorylated at various sites by different kinases. To investigate its phosphorylation more specifically, we conducted additional experiments with a reduced number of randomly selected adult allergic donors. Their BAT and pS6 results in pDCs and cDCs after stimulation are depicted in Supplemental Figure 1B. In pDCs, the increase of pS6 was most striking at the S235/S236 site, with only a minor increase at the S240 site and no detectable increase at the S244 site (Figure 1D). In cDCs, RPS6 was phosphorylated to a similar extent at the S235/S236 and S240 phosphorylation sites in whole blood stimulated with PN extract. However, the phosphorylation at S235/S236 was higher after stimulation with anti-IgE. Additionally, we detected a minor increase of phosphorylation at the S244 site only after anti-IgE stimulation, which might be caused by crosslinking of the FCεR1 receptors expressed at their surface. Therefore, we focused on staining pS6 at the S235/S236 site for all subsequent experiments.

### Stimulation with PN extract induces pS6 in allergic individuals but not in nonallergic individuals or basophil nonresponders.

Next, we analyzed the phosphorylation of RPS6 among peanut-allergic, nonallergic, and sensitized tolerant individuals (presence of peanut-specific IgE but no allergic reaction during the oral food challenge [OFC]) and in allergic basophils nonresponders (defined as individuals with <5% basophil activation upon stimulation with allergen or anti-IgE antibodies) (3). The age, sex, maximum tolerated dose at the OFC, and PN-specific IgE (PN-sIgE) mean are shown in Supplemental Table 2. Moreover, their basophil activation after stimulation with 10 μg/mL PN or 10 μg/mL anti-IgE antibody, as well as total IgE, PN-sIgE and Ara h 2-sIgE titers, are depicted in Supplemental Figure 1, D and E.

In nonallergic donors, pS6 levels in pDCs and cDCs increased only after stimulation with anti-IgE antibodies and were significantly lower in pDCs compared with peanut-allergic individuals (Figure 1E). These data suggest a peanut-specific IgE-dependent response, which is present at detectable levels only in sensitized individuals. Among sensitized-tolerant individuals, pS6 phosphorylation intensity in pDCs was lower than in allergic individuals, while phosphorylation levels in cDCs were comparable between the two groups. While pS6 induction exhibited a dose-dependent response in allergic individuals, it was induced in pDCs in sensitized-tolerant individuals only at 10 μg/mL PN. In both allergic and sensitized-tolerant individuals, the percentage of activated basophils followed a similar dose response (Supplemental Figure 1C). Moreover, peanut-sensitized basophil nonresponders exhibited lower pS6 induction after stimulation with anti-IgE antibodies or PN, although specific IgE titers of basophil nonresponders were comparable to those of allergic individuals. Altogether, this suggests that basophils contribute to pS6 induction.

### Degranulation of basophils is necessary for S6 phosphorylation in DCs.

Since the previous results suggested a mechanistic link between the PN-specific IgE and the increase of pS6 in DCs after stimulation with PN, we hypothesized that IgE and FCεR1 crosslinking followed by basophil degranulation and subsequent mediator release is required for this mechanism. However, both pDCs and cDCs express IgE receptors (10), and thereby, IgE binding may also directly contribute to pS6 induction. Moreover, we cannot exclude a direct uptake of the peanut protein by the DCs. To determine whether basophils are necessary for S6 phosphorylation in DCs, we magnetically depleted basophils from whole blood using anti-CCR3-biotin antibodies combined with either anti-biotin microbeads or PBS as control. Although this approach likely also depleted eosinophils that also express CCR3, eosinophils only express low levels of IgE high-affinity receptor and cell-bound IgE and do not degranulate upon IgE crosslinking (11). Therefore, it is unlikely that eosinophils are involved in the rapid increase of pS6 in pDCs and cDCs.

**Figure 2:**
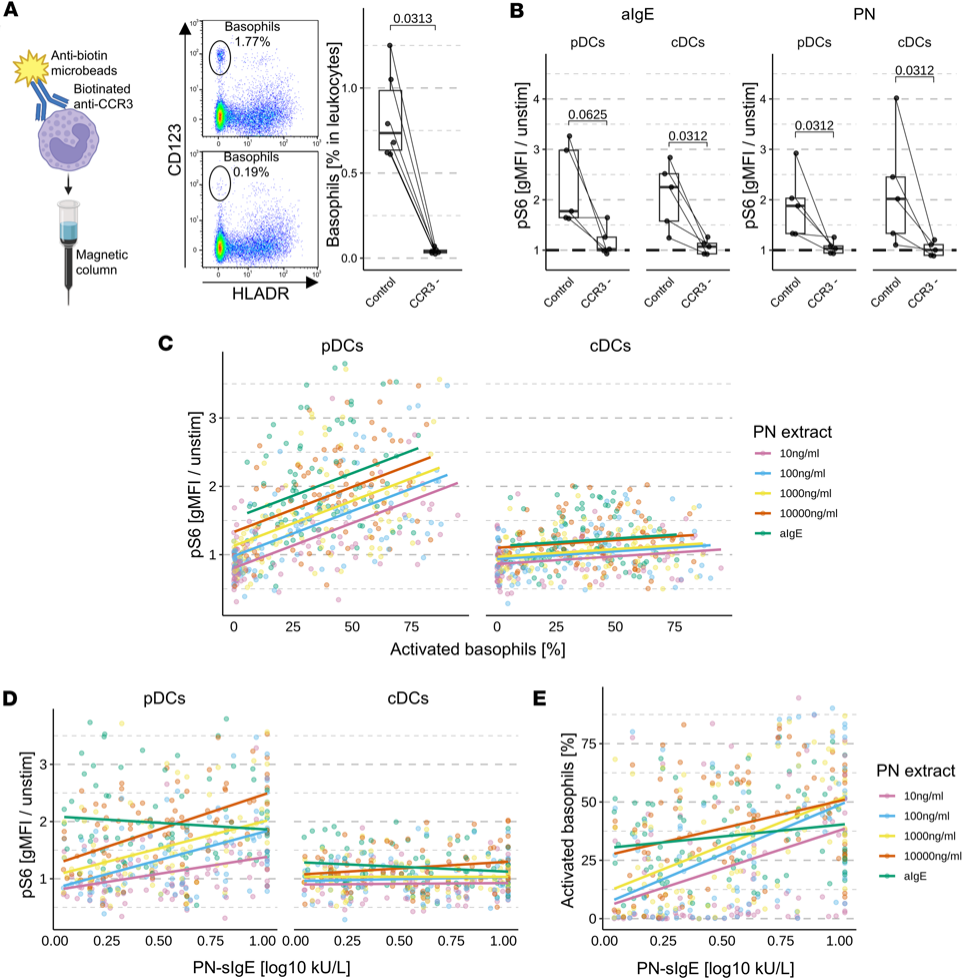
Basophil degranulation is necessary for the increase of pS6 in DCs. (**A**) Percentage of basophils in leukocytes after depletion of CCR3 cells from whole blood. (**B**) pS6 induction in whole blood with or without depletion of CCR3^+^ cells after stimulation with anti-IgE antibodies or PN extract (*n* = 5). Statistical tests were performed using paired Wilcoxon tests. *P* values are indicated. (**C**) Scatter plots depicting linear models of pS6 in pDCs and cDCs, with the percentage of activated basophils and different stimulations as explanatory variables (*n* = 114, 5 stimulations each). (**D**) Scatterplots depicting linear models of pS6 in pDCs and cDCs with levels of PN-sIgE (log_10_) and different stimulation as explanatory variables. (**E**) Plot showing the correlation between PN-sIgE (log_10_) and the percentage of activated basophils for different stimulations.

The depletion of CCR3^+^ cells did not affect percentages of DCs (Supplemental Figure 2A) but reduced basophil percentages by 10- to 20-fold (Figure 2A) and entirely inhibited pS6 upregulation in pDCs and cDCs in whole blood stimulated with 10 μg/mL anti-IgE antibodies or PN extract (Figure 2B).

### Degranulation of basophils and sIgE levels correlate with S6 phosphorylation in pDCs and cDCs.

Next, to confirm the role of basophil degranulation in the increase of pS6 in pDCs and cDCs in allergic and sensitized-tolerant individuals, we applied linear models using the percentage of activated basophils (scaled as decimal fractions for consistent slope estimates) and stimulation condition as explanatory variables (Table 1 and Table 2 for pDCs and cDCs, respectively). The generalized variance inflation factors (GVIFs) were lower than 2, indicating that no multicollinearity between the predictors was found. Residuals versus fitted and Q-Q plots of the models are displayed in Supplemental Figure 2A and reveal that the normality of the residual’s assumption was not fully met in the pDC model. Therefore, the calculated *P* values must be interpreted with caution. Correlation plots illustrating the models are shown for all stimulation conditions combined (Figure 2C) or separated by stimulation condition (Supplemental Figure 2C).

In pDCs and to a lesser extent in cDCs, we observed a significant correlation between pS6 and the percentage of activated basophils (*P* value < 2 × 10^-16^), thus further confirming their involvement in the increase of pS6 (Figure 2C). Interestingly, increasing the concentration of PN or using anti-IgE antibodies increased the level of pS6 independently of basophil activation, as indicated by the regression lines for the different stimulations, suggesting the involvement of other factors.

Therefore, we analyzed correlations between PN-sIgE level and pS6 in blood stimulated with different concentrations of PN extract or anti-IgE (Figure 2D shows all stimulation conditions combined, while Supplemental Figure 2D shows the stimulation conditions separately). Notably, PN-sIgE levels correlated with pS6 in blood stimulated with PN in pDCs and at 1 or 10 μg/mL in cDCs. These observations may reflect the crosslinking of sIgE and subsequent signaling through the high- or low-affinity IgE receptor. As expected, no correlation was observed with aIgE-stimulated blood, since this positive control does not rely on specific antibodies.

However, PN-sIgE levels also correlated with the percentage of activated basophils (Figure 2E for all stimulation conditions combined, and Supplemental Figure 2E for separated conditions). To disentangle the effect of activated basophils and PN-sIgE on pS6, we further computed linear models additionally including PN-sIgE titers. Because the slopes of the correlations between PN-sIgE levels and pS6 differed across different concentrations of PN extract, we added an interaction term between these two explanatory variables. Estimates of the contribution of the explanatory variables on the increase of pS6 in pDCs and cDCs are displayed in Table 3 and Table 4, respectively. Residuals versus fitted and Q-Q plots of the models are displayed in Supplemental Figure 2B.

In line with the previous model, we observed a correlation between PN-sIgE levels and pS6 in pDCs at PN concentrations of 100 ng/mL and higher, while the correlation between activated basophils and pS6 remained unchanged. In cDCs, a moderate but nonsignificant effect of PN-sIgE on pS6 was observed only at a high concentration of 10 μg/mL PN extract. Interestingly, the contribution of PN-sIgE was more pronounced in pDCs than in cDCs, despite the latter expressing higher levels of FCεR1 (10). This discrepancy might be attributed to the generally weaker induction of pS6 in cDCs. In addition, adding PN-sIgE levels to the linear model greatly mitigated the contribution of the different concentrations of PN on pS6 induction, potentially explaining the observed effects. For instance, adding PN-sIgE titers to the model reduced the estimated contribution of stimulation with 10 μg/mL PN extract (compared with 10 ng/mL) in pDCs from 0.53 (Table 1) to 0.24 (Table 2).

Together, these findings confirmed that basophil activation and degranulation play a critical role in pS6 induction in pDCs and cDCs, with PN-sIgE providing an additional contributory effect.

### Blocking the H2R abolished the induction of pS6 in pDCs and cDCs.

**Figure 3:**
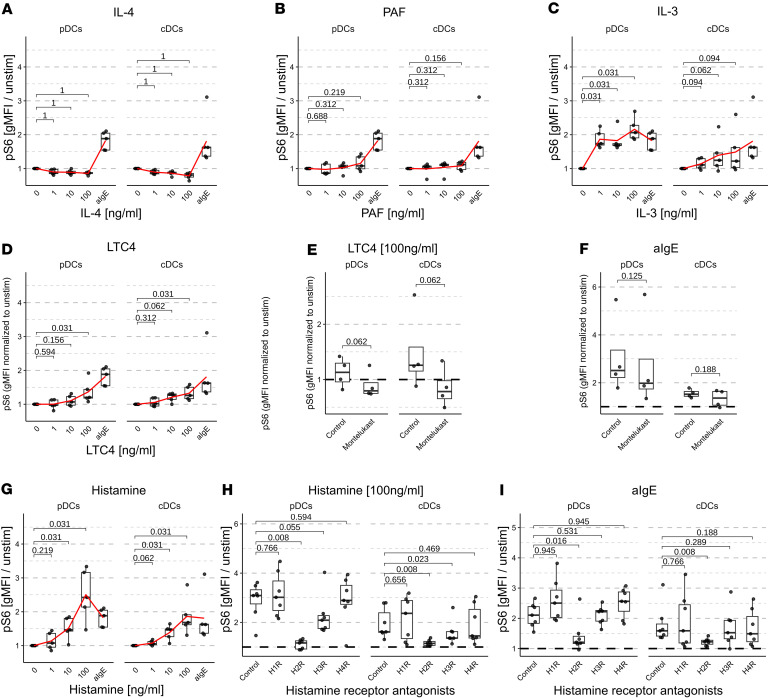
Histamine signaling through H2R is necessary for the phosphorylation of RPS6 in DCs after stimulation of whole blood with anti-IgE antibodies. (**A**–**D** and **G**) pS6 in pDCs or cDCs after stimulation of whole blood with 0, 1, 10, and 100 ng/mL of the indicated mediators or 10 μg/mL anti-IgE (*n* = 5). (**E** and **F**) pS6 gMFI in whole blood preincubated with 10^–5^ M Montelukast, a LTC4 receptor antagonist, and further stimulated with 100ng/ml LTC4 or anti-IgE antibodies (*n* = 4). (**H** and **I**) pS6 gMFI in whole blood preincubated with 10-5 M H1R, H2R, H3R and H4R antagonists (Fexofenadine, Famotidine, Pitolisant, and JNJ7777120 respectively), and further stimulated with 100ng/ml histamine or anti-IgE antibodies (*n* = 7). All statistical tests were performed using 1-tailed paired Wilcoxon’s tests. *P* values are indicated.

To identify which mediators released by basophils contribute to the in vitro phosphorylation of S6 in DCs, we investigated several basophil-derived mediators at different concentrations in our assay. Neither IL-4 nor PAF had a notable effect on pS6 in DCs (Figure 3, A and B). At 1 ng/mL and above, IL-3 increased pS6 in pDCs at a similar level to anti-IgE. In cDCs, pS6 levels also increased with IL-3 stimulation, though even the high dose of 100 ng/mL failed to achieve the levels observed with anti-IgE antibody stimulation (Figure 3C). However, the rapid induction of pS6 observed within 30 minutes of the stimulation and coinciding with basophil degranulation (Supplemental Figure 3A) suggests that preformed and rapidly secreted mediators rather than cytokines expressed after activation were responsible for the increase of pS6. This is supported by the evidence that IL-3 mRNA is not constitutively expressed in basophils and reaches its peak of expression only 60 minutes after stimulation (12). Blocking IL-3 receptors did not sufficiently prevent the phosphorylation of RPS6 after stimulation with IL-3, especially in the pDCs (data not shown). Therefore, we could not entirely rule out the contribution of IL-3 in the increase of pS6.

LTC4 induced a slight increase of pS6 in pDCs and cDCs, but the levels did not reach those observed with anti-IgE antibody stimulation (Figure 3D). To further assess the contribution of LTC4 in the increase of pS6, we blocked the LTC4 receptor using Montelukast, which effectively reduced the pS6 induction by LTC4 (Figure 3E). However, blocking LTC4 receptors did not reduce the pS6 increase in whole blood stimulated with anti-IgE antibodies (Figure 3F) or PN (Supplemental Figure 3C), indicating that LTC4 does not play a role in the studied mechanism.

Histamine, a preformed mediator stored in the basophilic granules of basophils, was shown to exert various effects on DCs, which express the histamine 1, 2, and 4 receptors (H1R, H2R, and H4R, respectively), while the expression for H3R is more controversial (13). Indeed, histamine induced a dose-dependent increase in pS6 in both pDCs and cDCs (Figure 3G). To confirm its involvement, we used antagonists specific for histamine receptors H1–H4: Fexofenadine (H1), Famotidine (H2), Pitolisant (H3), and JNJ7777120 (H4). Among these, only Famotidine, the H2 receptor antagonist, completely abolished pS6 induced by histamine (Figure 3H), anti-IgE antibodies (Figure 3I), and PN (Supplemental Figure 3D). The H3 antagonist resulted in a smaller reduction of pS6 induction in pDCs and cDCs stimulated with histamine, while showing no significant effect in cells stimulated with aIgE antibodies and only a minor decrease in pS6 induction in cDCs exposed to PN. This is likely due to the cross-reactivity and unspecific blocking of the H2 receptor. Interestingly, blocking H1 and H4 resulted in increased pS6 levels in pDCs stimulated with PN, highlighting their inhibitory role in pS6 by histamine. Notably, the H4 pathway depends on the Gi protein (14), which inhibits cAMP and consequently reduces pS6. Blocking H1R may enhance the amount of histamine binding of H2R, thereby increasing pS6 levels. Furthermore, we examined the kinetic of pS6 induction following stimulation with 100 ng/mL histamine. In both pDCs and cDCs, pS6 levels increased as early as 5 minutes, peaked between 30 and 60 minutes and declined until 240 minutes (Supplemental Figure 3E). In contrast, when histamine was removed by 2 washes with media, pS6 induction was already lower at 15 minutes and was absent at 30 minutes after washing (Supplemental Figure 3F), suggesting that phosphorylation of S6 through histamine is even more transient, i.e., limited to approximately 15 minutes.

Together with the previous findings, this demonstrated that histamine released by basophils upon crosslinking of their FcεRI receptors signals through the H2 receptor in pDCs and cDCs, driving the phosphorylation of RPS6.

### In adults, the increase of pS6 in cDCs is associated with the severity of the allergic reaction.

**Figure 4:**
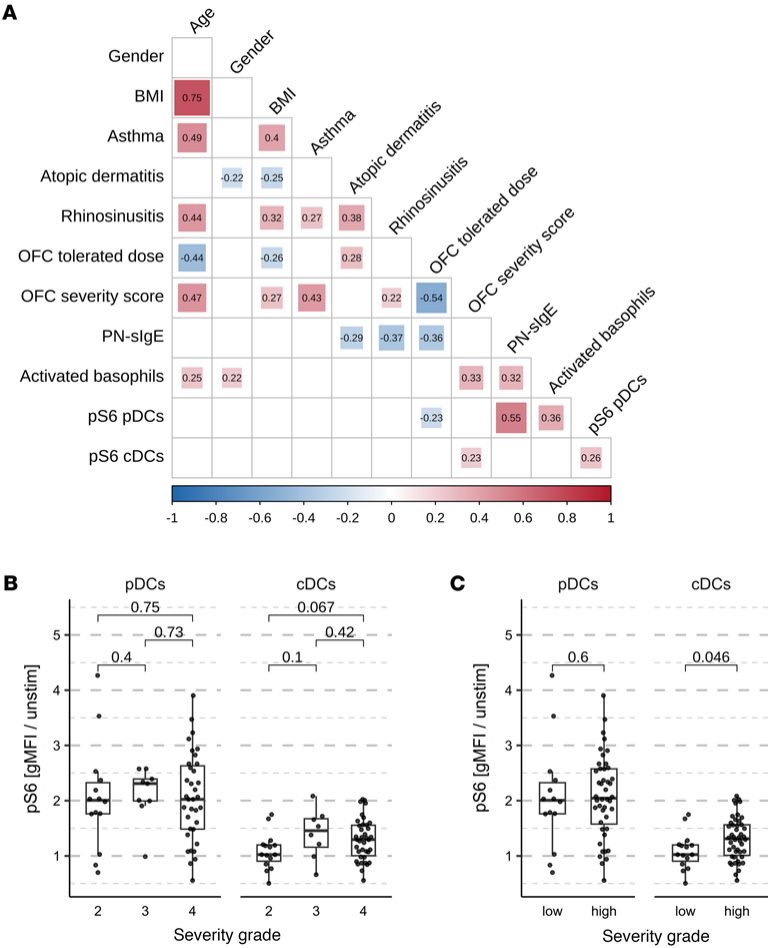
RPS6 phosphorylation in cDCs is associated with the allergic reaction severity. (**A**) Correlation matrix in PN allergic donors between indicated clinical parameters and pS6 in pDCs and cDCs after stimulation with 10 μg/mL PN extract. Only correlations with *P* < 0.1 are displayed. (**B**) Increase of pS6 (gMFI was normalized to that in unstimulated blood) in pDCs and cDCs with different severity grades determined during an OFC and (**C**) categorized into low (grade 2) versus high (grade 3 and 4 together) groups (*n* = 61). The statistical tests were performed using Wilcoxon’s tests. *P* values are indicated.

To investigate the association between clinical parameters and the increase of pS6 in pDCs and cDCs among allergic donors, we generated a correlation matrix of various clinical parameters and pS6 increase in pDCs and cDCs after stimulation with 10 μg/mL PN extract (Figure 4A). The included donors were between 2 and 40 years old (mean age 19.5 years), had a positive OFC to peanut (performed after blood collection), and exhibited basophil degranulation in response to positive control. Notably, we identified an association between increased pS6 in cDCs, but not pDCs, and the severity of the allergic reaction determined during the oral food challenge for peanuts using the modified Sampson score (15). When plotting severity against pS6 levels in cDCs, two distinct patterns emerged: individuals with a severity grade of 2 showed no increase in pS6 after stimulation with 10 μg/mL PN extract, whereas those with severity grades of 3 or 4 exhibited similar increases in pS6 (Figure 4B). To ensure a linear correlation for subsequent models, we categorized severity grades into 2 groups: low (grade 2) and high (grades 3 and 4) (Figure 4C).

The severity grade was also associated with age and with the percentage of activated basophils. While pS6 levels in cDCs did not initially appear to correlate linearly with age (Supplemental Figure 4A), stratifying the data by age groups revealed a nonlinear relationship: pS6 levels increased during childhood and peaked around 20 years of age, followed by a decline in older individuals (Supplemental Figure 4B). Adults, on average, exhibited a stronger but not significant pS6 increase in cDCs (Supplemental Figure 4C). The association of the severity grade with BMI and asthma may likely be secondary effects of the strong correlation with age in our dataset.

Therefore, to account for variations in basophil activation across the different severity grades, we computed another linear model with the increase of pS6 in cDCs as the response variable and the age, percentage of activated basophils, PN-sIgE titers, and the severity score (low or high) in children or in adults as explanatory variables (Table 5). Residuals versus fitted and Q-Q plots are displayed in Supplemental Figure 4D, and the age, sex, maximum tolerated dose at the OFC, and the PN-sIgE titers are shown in Supplemental Table 3. This unveiled a significant association between the severity and pS6 in cDCs but only in adults. Specifically, adults with high-severity allergic reactions exhibited a 0.46 greater increase in pS6 compared with those with low-severity reactions (*P* value = 0.003). No such associations were observed in children. Additional added variable plots depicting pS6 induction in children or adults of low or high severity grade are shown in Supplemental Figure 4E. Interestingly, similar results were obtained in blood stimulated with anti-IgE antibodies (linear model in Supplemental Table 4), further supporting the conclusion that the differential increase in pS6 in adults with severe allergic reactions arises due to cellular rather than serological factors.

## Discussion

Herein, we investigated the phosphorylation of RPS6 at serine S235/S236 in pDCs and cDCs (cDC1 and cDC2 combined) following whole-blood stimulation with increasing concentrations of PN or anti-IgE antibodies as positive control. We detected strong and weaker pS6 induction in pDCs and cDCs, respectively, but not in monocytes, which may be due to a lower sensitivity of fluorescence-based flow cytometry compared with the mass cytometric method presented by Tordesillas et al (6). Stimulation with PN extract led to increased pS6 levels only in PN-allergic donors. Similarly, stimulation with anti-IgE antibodies induced equivalent basophil activation but higher pS6 levels in pDCs of allergic donors compared with nonallergic donors, which may be attributable to higher total IgE levels in allergic donors. Additionally, pS6 levels were almost absent in basophil nonresponder individuals, suggesting that basophil degranulation after PN-sIgE and FCεR1 crosslinking mediates pS6 in DCs.

This was confirmed by depleting CCR3^+^ cells from whole blood, including basophils (16), eosinophils (17), and a small subset of T cells (18), which abolished the increase of pS6 in both pDCs and cDCs. Although contributions from other CCR3^+^ cells cannot be entirely excluded, the correlation between basophil activation and pS6 levels, coupled with the absence of pS6 increase in basophil nonresponders, strongly implicates basophil activation and degranulation as the primary mechanism. Moreover, we identified a correlation between PN-sIgE level and the increase of pS6 in pDCs and cDCs, suggesting a secondary effect of the crosslinking of PN-sIgE at the surface of the DCs. Membrane-bound IgEs are unlikely to play a role as they are absent in pDCs (19) and have not been described in cDCs. We could not rule out an additional contribution of direct allergen uptake by DCs or the binding of other receptors independently of IgE to the induction of pS6. Finally, histamine induced pS6 in pDCs and cDCs, and blocking H2R using an antagonist abolished the increase of pS6 in pDCs and cDCs induced by histamine, anti-IgE antibody, or PN. Because eosinophils or T cells do not release histamine, this highlights the primary role of basophils in the observed increase of pS6. Although strong evidences of the benefit of H2R antagonists on the prevention of anaphylaxis reactions are lacking (20), the EAACI guidelines recommended them as third-line treatment (21) based on their capacity to improve cutaneous symptoms (22, 23). Furthermore, a recent study suggested a benefit of Famotidine on hypersensitivity reactions to platinum or taxane-based chemotherapies (24). Together with our results, this suggests potential but controversial beneficial effects of H2R antagonists on anaphylaxis reactions.

DCs express the protease-activated receptor 2, which can be triggered by tryptase, a mediator released by mast cells, especially in severe allergic reactions. Since basophils express only very low levels of tryptase compared with mast cells, we did not assess the contribution of this mediator to the induction of pS6 and cannot rule out an effect on pS6 in addition to histamine.

pDCs play a crucial role in controlling viral infection by secreting type 1 interferons and presenting antigens to CD8^+^ and CD4^+^ T cells. Their role in the immune response to food allergies remains unclear. However, they were shown to promote oral tolerance and protect against allergic airway inflammation in mice (25, 26) and were associated with tolerance toward peanut in children (27). Future studies investigating the role of the H2R and RPS6 on the function and potential tolerogenic capacities of pDCs are required.

cDCs can be further separated in different subsets such as cDC1 and cDC2. In the peripheral blood, cDC2 is the dominant subset and can induce Th2 and Th17 skewing. Histamine is a key mediator in early- and late-phase allergic reactions, signaling through histamine receptors on immune cells (28). Notably, H2R signaling enhances antigen uptake and cross-presentation (29), decreases the IFN-α and TNF-α produced by pDCs (30), and promotes Th2-skewing DC polarization (31, 32). Furthermore, histamine has been shown to prevent the in vitro apoptosis of monocytes through H2R and cAMP signaling (33). H2R is a G protein–coupled receptor signaling through the cAMP/PKA pathway (28), which can then phosphorylate RPS6 at the S235/S236 site (34). Although its role in antigen presentation remains unclear, pS6 promotes cell growth, migration, and apoptosis prevention (8), potentially enhancing antigen-presenting cell functions and contributing to the persistence of allergic responses. We observed a fast increase of pS6 that peaked between 30 and 60 minutes after stimulation with histamine, which is in line with the timing of antigen uptake and presentation (35). We were unable to determine the factors underlying the greater increase of pS6 observed in pDCs compared with cDCs. This may stem from a stronger response to histamine via H2R and the cAMP/PKA pathway in pDCs or from a more tightly regulated phosphorylation of RPS6 in cDCs.

Finally, we identified an age-dependent association between the severity of allergic reactions and the increase in pS6 levels in cDCs. Specifically, allergic adults — but not children — with higher severity grades during oral food challenges exhibited greater pS6 phosphorylation in cDCs. However, the observed difference in pS6 induction is not strong enough to delineate between severity groups and thus cannot be used alone as a predictive biomarker for allergic severity. Furthermore, these findings should be replicated in greater cohort studies, particularly in other food allergies, to determine whether the observed mechanisms are consistent and can be generalized. The mechanisms leading to higher phosphorylation of RPS6 in adults with severe allergic reactions as well as its biological implications remain to be elucidated. This phenomenon is unlikely to result from differences in histamine production by basophils, as no variations in pDC responses were observed between groups. Instead, age- and cell-type-specific changes in histamine receptor expression or downstream signaling pathways, such as the cAMP/PKA pathway, may contribute to this effect. Elevated pS6 in cDCs could enhance antigen presentation by promoting greater cDC survival and mobility. This may perpetuate the impaired immune tolerance observed in allergic individuals, especially adults, by reinforcing type 2 immune responses and contributing to more severe allergic reactions. In such cases, pS6 could serve as a valuable biomarker for assessing the activation of this pathway in the clinic, as it can be measured together with the BAT.

To conclude, this study reveals that histamine released by basophils signals in pDCs and cDCs through H2R, leading to pS6. This process is associated with both age and the severity of allergic reactions. These findings support the dual H1R and H2R blockade or the modulation of the cAMP/PKA pathway in antigen-presenting cells as a potential treatment strategy, as suggested by others (36, 37). Further research on the effect of differential pS6 induction on DC function is needed to clarify the underlying mechanisms and their clinical relevance.

## Methods

### Sex as a biological variable.

Both male and female individuals were analyzed in this study. Sex was considered as a variable in our models, but it was then removed as it did not play a significant role.

### Sampling.

Blood was collected in Vacuette lithium heparin or in Vacuette serum tubes (Greiner). For the modified BAT, blood was used within 6 hours of blood collection.

### PN.

The allergen extract was prepared as described previously (38).

### Determination of serum antibody titers.

Serum was analyzed using the ImmunoCap Phadia 200 immunoassay according to the manufacturer’s instructions (Thermo Fisher Scientific). The determination of all specific antibodies was based on a full PN (f13) as well as on the peanut-specific allergen component Ara h 2 (f423). The cutoff level for the sIgE was set at 0.35 kU/L. The detection of sIgE and total IgE included the additional application of several quality controls (low, medium, and high concentrated).

### CCR3-expressing cell depletion.

Whole blood was incubated with CCR3-biotinylated antibody (Miltenyi) at optimum titration for 10 minutes at room temperature. Next, PBS (control) or anti-biotin microbeads (Miltenyi) were added and incubated further for 10 minutes at room temperature. The samples were processed using MS columns (Miltenyi), and the negative fraction was collected directly without washing to avoid further dilution of the blood sample.

### BAT.

40 μL of whole blood was incubated at 37°C, 5% CO_2_ for 30 min (or for 5 min, 15 min, and 30 min when indicated) with PBS; 10 μg/mL anti-IgE antibodies (goat polyclonal, Thermo Fisher Scientific); 10 μg/mL PN (or 10 ng/mL, 100 ng/mL, 1 μg/mL, and 10 μg/mL when indicated); and 10^–5^ M fMLP (Sigma-Aldrich) or 1, 10, and 100 ng/mL histamine (Sigma-Aldrich), LTC4 (Biomol), IL-3 (Miltenyi), IL-4 (Miltenyi), or platelet-activating factor 16 (Calbiochem). For blocking experiments, whole blood was preincubated before the stimulation for 15 minutes at 37°C with 10 μM LTC4 receptor antagonist Montelukast (Sigma Aldrich) or 10 μM histamine receptor antagonists Fexofenadine (Sanofi Aventis), Famotidine (Sigma Aldrich), Pitolisant (Sigma Aldrich), or JNJ7777120 (Johnson and Johnson).

During the incubation with stimulants, surface staining was performed in the presence of 1 mg/mL beriglobin (CSL Behring) to block unspecific antibody binding. The list of antibodies used for staining can be found in Supplemental Table 1. Stimulations were stopped and samples were fixed by incubation with 20 nM EDTA and BD FACS lysing solution (BD Biosciences) for 10 min. Staining post-fixation was performed for 20 min, and the samples were permeabilized with BD Phosphoflow Perm Buffer II (BD Bioscience) according to the manufacturer’s protocol. Intracellular staining was carried out for 30 min in the dark at room temperature.

### Flow cytometry measurement and analysis.

All samples were measured on a MACSQuant Analyzer 16 (Miltenyi) according to the gating strategy illustrated in Supplemental Figure 1A. Instrument performance was monitored before every measurement with Rainbow Calibration Particles (BD). Flow cytometry data were analyzed using FlowJo 10 (BD).

### Statistics.

Clinical data were collected and managed using REDCap electronic data capture tools hosted at Charité. The clinical data and laboratory results were compiled and processed, and all statistical analyses were calculated using R (version 4.4.0) and RStudio (2024.04.1+748). Samples with abnormal flow cytometric staining due to technical issues were excluded from the analysis. Since the data were nonnormally distributed, nonparametric comparisons were performed (paired or nonpaired Wilcoxon’s test and Spearman’s correlation test). Results of statistical comparisons are displayed as *P* values, and a *P* value of less than 0.05 was considered significant.

### Study approval.

Eligible participants were part of the randomized clinical trial TINA (Tolerance induction through nonavoidance to prevent persistent food allergy; trial ID DRKS00016764), which is currently conducted at the Charité – Universitätsmedizin Berlin. The study was approved by the local ethics committee (Charité – Universitätsmedizin Berlin) (EA2/033/19 and EA2/032/19). Participants were recruited following the principles of the Declaration of Helsinki from the Division of Allergy and Immunology, Department of Dermatology, Venereology and Allergy, Campus Mitte and from the Division of Pediatric Allergy, Department of Pediatric Respiratory Medicine, Immunology and Critical Care Medicine, Campus Virchow-Klinikum, with a known peanut allergy (allergic) or no food allergy (nonallergic). Peanut-sensitized donors were classified as sensitized-allergic or sensitized-tolerant based on the result of an oral food challenge performed after sample collection.

### Data availability.

Values for all data points presented in plots and in linear models are reported in the Supporting Data Values file. All results and flow cytometry data will be made available upon request.

## Author contributions

FF planned and performed experiments and analyzed the data. JB, MW, and AT designed and supervised the study. FF and JB wrote the manuscript.

## Funding support

This work was funded by the DFG, Deutsche Forschungsgemeinschaft (German Research Foundation), as part of the clinical research unit (CRU339), FOOD@, project no. 409525714.

## Supplementary Material

Supplemental data

Supporting data values

## Figures and Tables

**Table 1:**
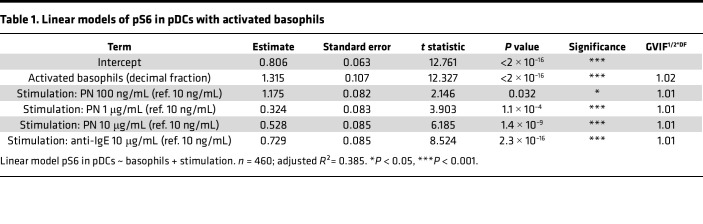
Linear models of pS6 in pDCs with activated basophils

**Table 2:**
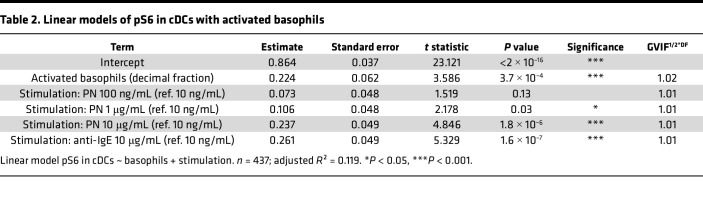
Linear models of pS6 in cDCs with activated basophils

**Table 3:**
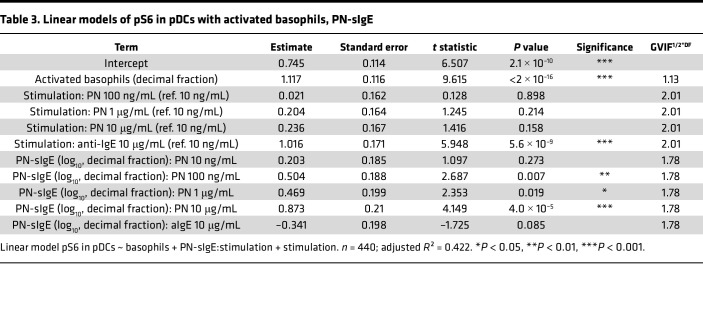
Linear models of pS6 in pDCs with activated basophils, PN-sIgE

**Table 4:**
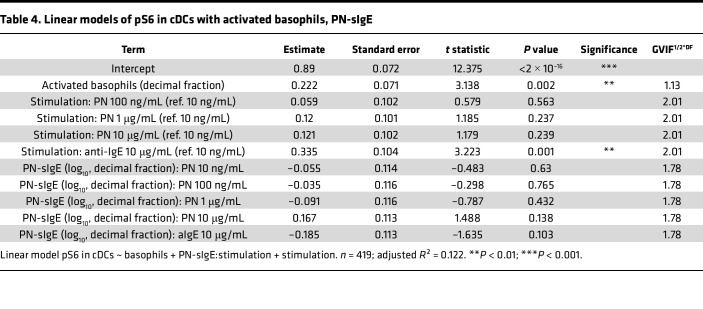
Linear models of pS6 in cDCs with activated basophils, PN-sIgE

**Table 5:**
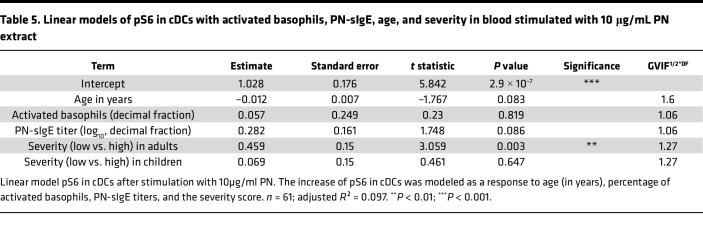
Linear models of pS6 in cDCs with activated basophils, PN-sIgE, age, and severity in blood stimulated with 10 μg/mL PN extract

## Version 1 09/11/2025

In-Press Preview

## Version 2 10/22/2025

Electronic publication
