# Supplementary material for: Histamine-induced RPS6 phosphorylation in dendritic cells is associated with the severity of peanut allergic reactions
Source: JCI Insight. 2025 Sep 11;10(20):e196167. doi: 10.1172/jci.insight.196167 (PMC12581671; doi:10.1172/jci.insight.196167)
Supplement: Supplemental data [file jciinsight-10-196167-s190.pdf]

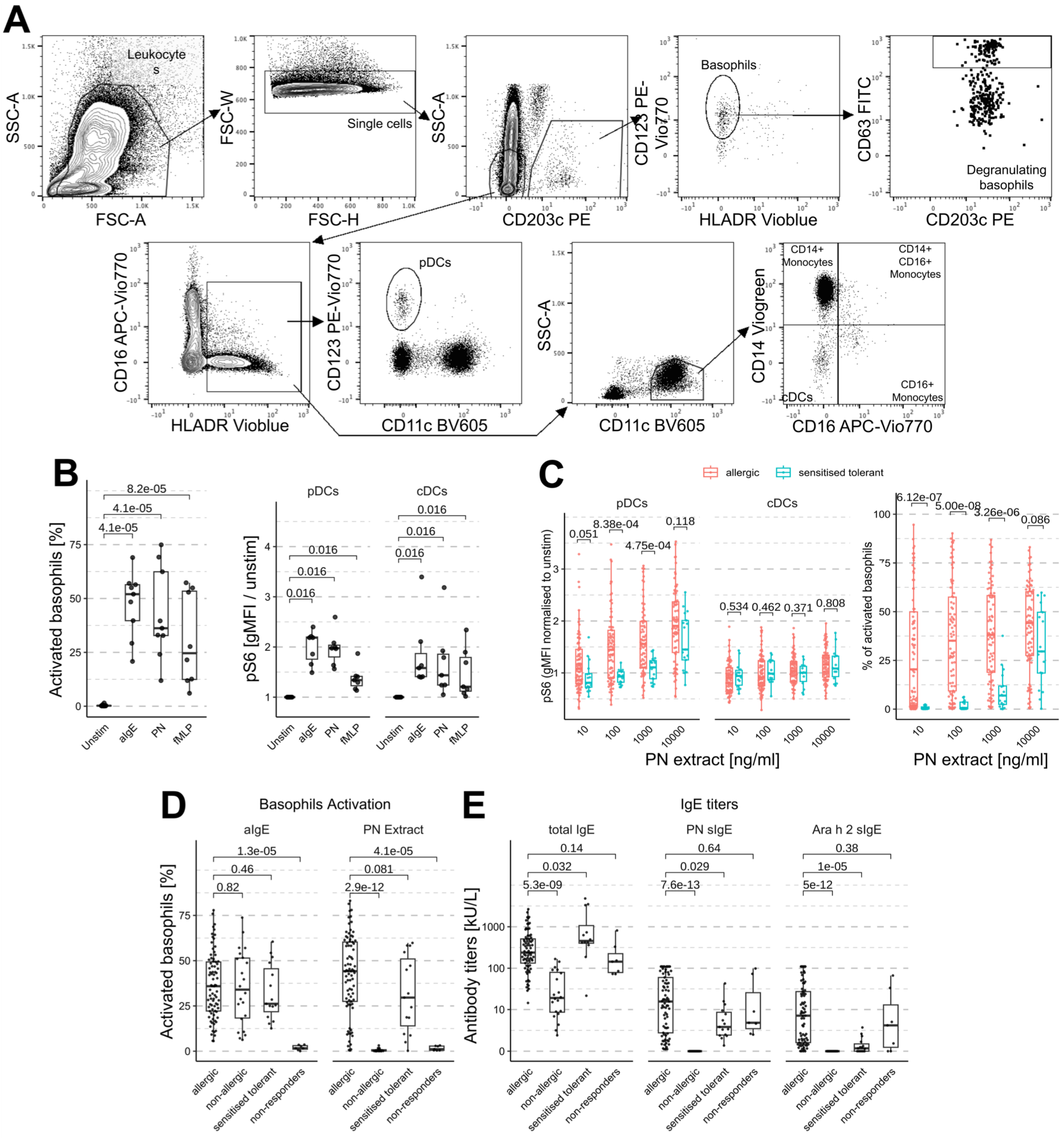

**Supplementary Figure 1: A:** Flow cytometry gating strategy. **B:** Activated basophils and RPS6 phosphorylation in pDCs and cDCs in the samples used for the mechanistic assessments. Statistical tests were performed using paired Wilcoxon tests. P-values are indicated. **C:** pS6 induction and percentage of degranulating basophils (CD63 high) in allergic and sensitised tolerant with stimulation with different concentrations of PN extract. **D:** Percentage of activated basophils and **E:** titer of total IgE, PN-sIgE and Ara h 2-sIgE in kU/L in PN-allergic, non-allergic, PN-sensitised but tolerant and PN-allergic basophils non-responder donors after stimulation with 10µg/ml PN-extract or anti-IgE antibodies. Statistical tests were performed using non-paired Wilcoxon tests. P-values are indicated.

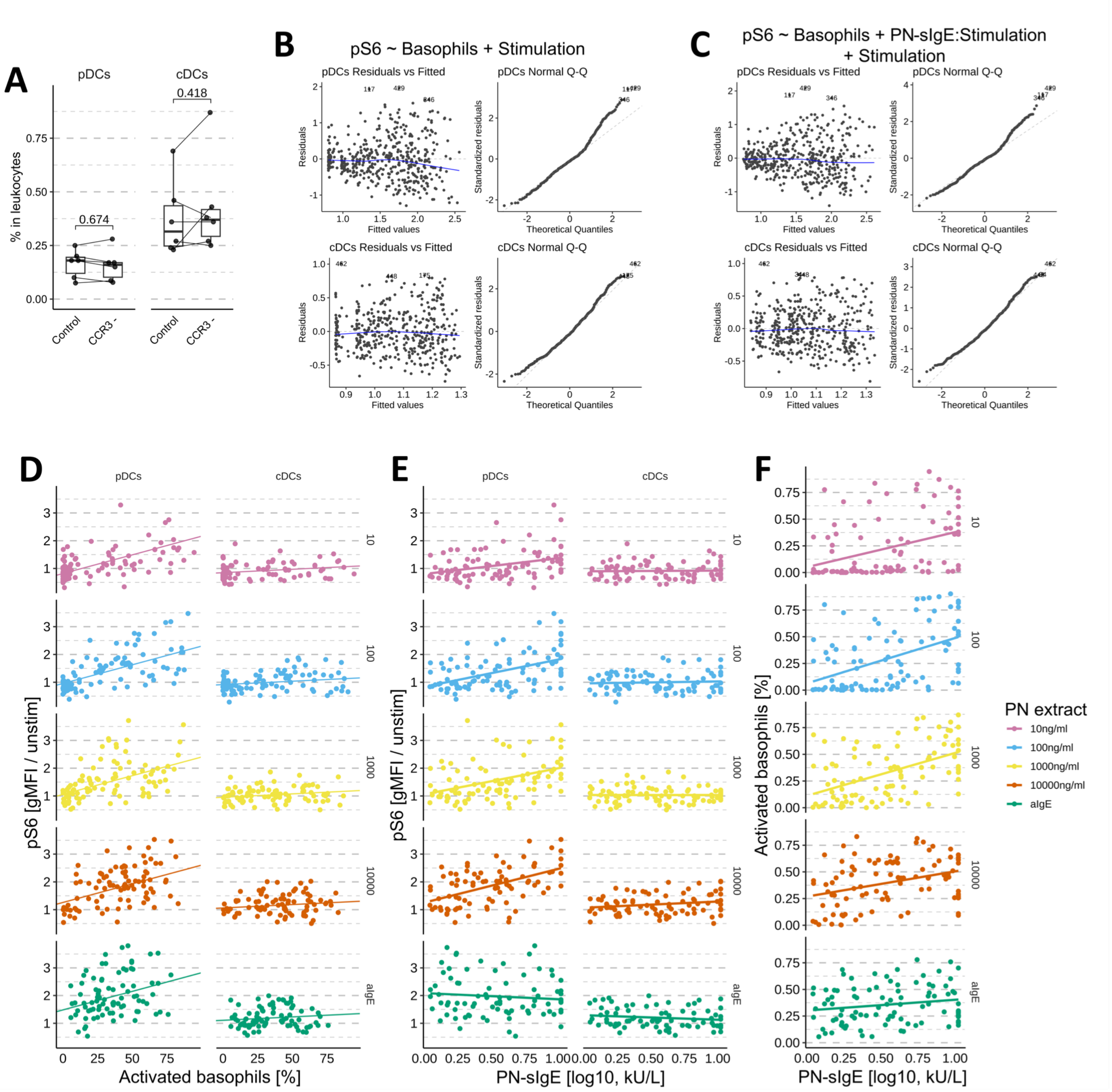


**Supplementary Figure 2:**

**A:** Percentage of pDCs and cDCs in leukocytes after depletion of CCR3 cells from whole blood. **B-C:** Residuals vs Fitted and normal Q-Q plot of the linear model of pS6 in pDCs and cDCs with **B)** Percentage of activated basophils and stimulation **C)** Percentage of activated basophils, PN-sIgE and stimulation as explanatory variables. **D:** Scatter plots depicting linear models of pS6 in pDCs and cDCs with the percentage of activated basophils and different stimulation as explanatory variables. **E:** Scatter plots depicting linear models of pS6 in pDCs and cDCs with levels of PN-sIgE (log 10) and different stimulation as explanatory variables. **F:** Plot showing the correlation between PN-sIgE (log 10) and the percentage of activated basophils for different stimulations.

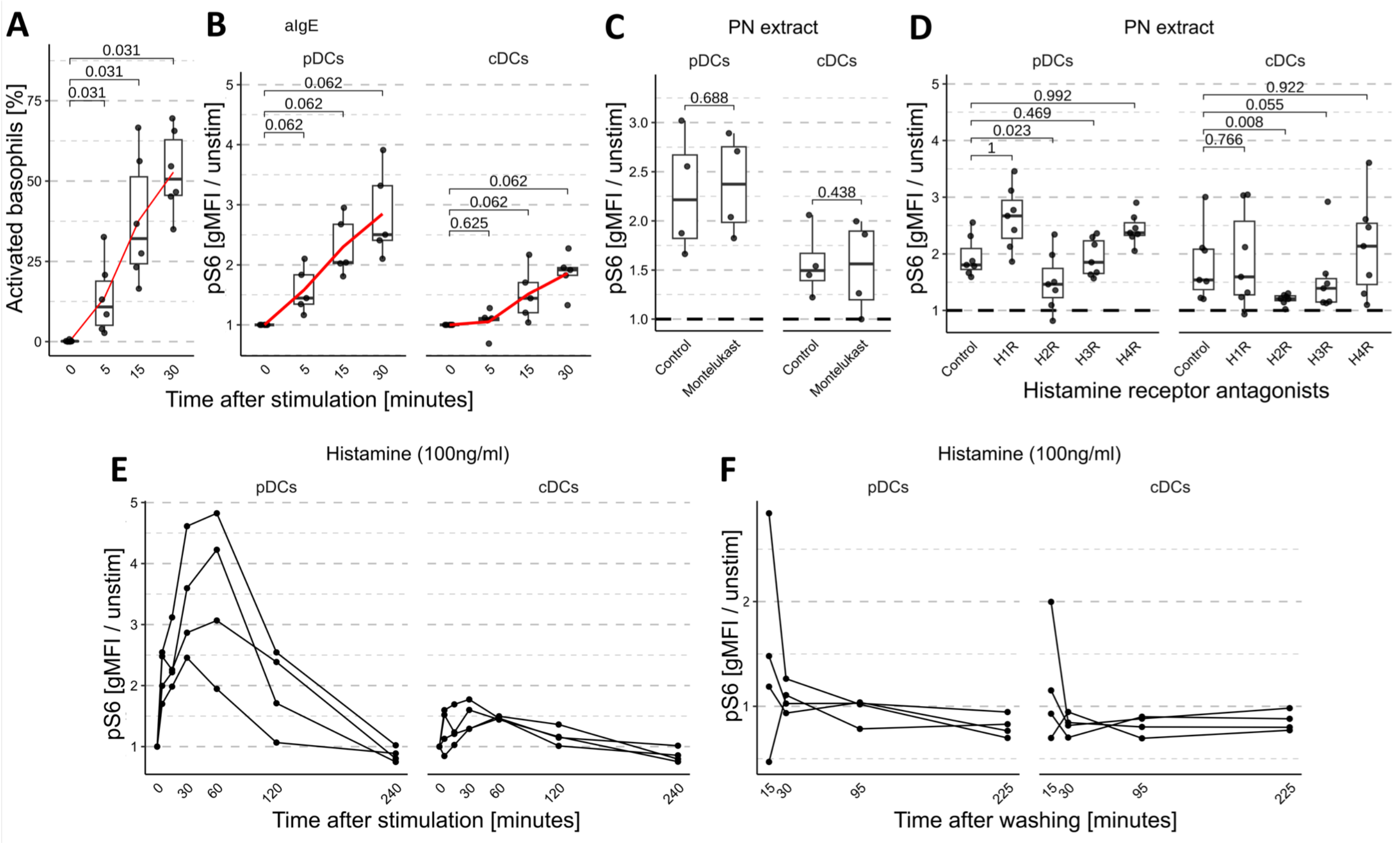


**Supplementary Figure 3: A:** Percentage of activated basophils and **B:** pS6 (gMFI normalised to unstimulated) in pDCs and cDCs after stimulation with anti-IgE antibodies for 0 (unstimulated), 5, 15 or 30 minutes. **C:** pS6 (gMFI normalised to unstimulated) in pDCs and cDCs after stimulation of whole blood with PN extract after incubation with $10^{-5}$M Montelukast. **D:** pS6 (gMFI normalised to unstimulated) in pDCs and cDCs after stimulation of whole blood with PN extract after incubation with $10^{-5}$M of H1R, H2R, H3R and H4R antagonists (Fexofenadine, Famotidine, Pitolisant and JNJ7777120, respectively). The statistical tests were performed using paired (A, B) or one-tailed paired Wilcoxon tests (C, D). P-values are indicated. **E:** pS6 (gMFI normalised to unstimulated) in pDCs and cDCs after stimulation with 100ng/ml histamine for 0 (unstimulated), 5, 15, 30, 60, 120 or 240 minutes and **F:** after 15, 30, 95, 225 minutes after stimulation with 100ng/ml histamine for 30 minutes and washing of the histamine.

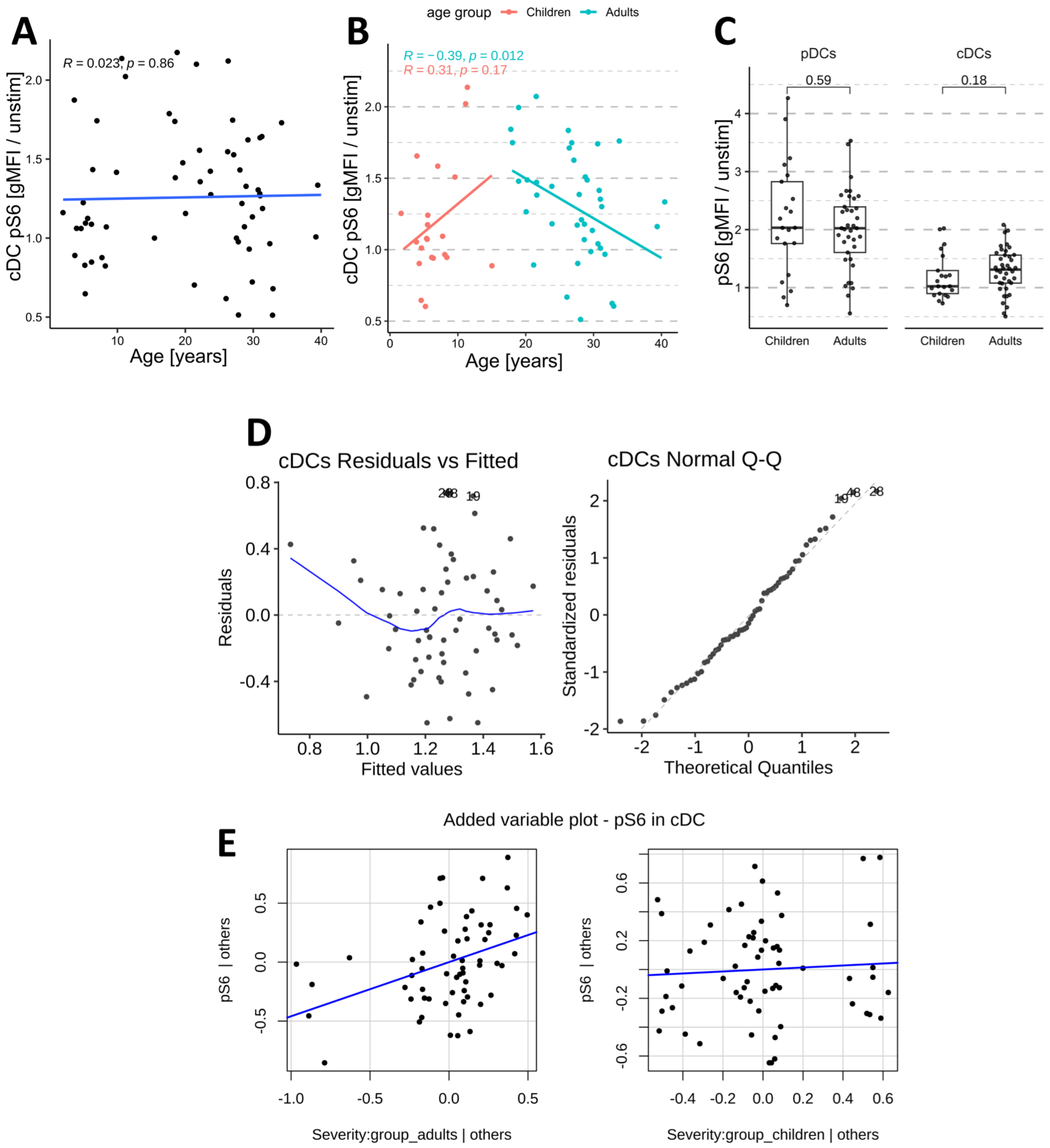


**Supplementary figure 4**

**A:** Correlation between pS6 (gMFI normalised to unstim) in cDCs and age in year and **B:** with separated correlation between children and adults. **C:** Increase of pS6 (gMFI normalised to unstim) in pDCs and cDCs in children compared to adults. **D:** Residuals vs Fitted and normal Q-Q plot of the linear model of pS6 in cDCs with the age in years, percentage of activated basophils, PN-sIgE titer and severity interacting with the age group (children or adults). E: Added variable plot of pS6 (gMFI normalised to unstimulated and normalised for the other variables included in the model) vs the severity score (low vs high) in adults or in children.

## Table S1: Antibody panel

| Marker | Fluorophore | Clone | Manufacturer | Catalog number |
|---|---|---|---|---|
| HLADR | Vioblue | REA805 | Miltenyi biotech | 130-111-794 |
| CD14 | Viogreen | TÜK4 | Miltenyi biotech | 130-113-153 |
| CD11c | BV605 | 3.9 | Biolegend | 301636 |
| CD63 | FITC | REA1055 | Miltenyi biotech | 130-118-076 |
| CD203c | PE | REA826 | Miltenyi biotech | 130-112-624 |
| CD123 | PE-Vio770 | REA918 | Miltenyi biotech | 130-115-266 |
| S6 p265/236 | APC | REA454 | Miltenyi biotech | 130-124-255 |
| S6 p240 | APC | REA420 | Miltenyi biotech | 130-117-372 |
| S6 p244 | APC | A18024A | Biolegend | 935706 |
| CD16 | APC-Vio770 | REA423 | Miltenyi biotech | 130-113-390 |

## Table S2: Selected clinical information for various patient cohorts

| Cohorts | Allergic (n=73) | Non-allergic (n=18) | Sensitised tolerant (n=14) | Non-responder (n=6) | Total (n=111) |
|---|---|---|---|---|---|
| Male proportion | 0.42 | 0.50 | 0.64 | 0.50 | 0.47 |
| Age mean (SD) | 18.59 (10.79) | 30.78 (7.31) | 17.93 (12.12) | 8.50 (11.10) | 19.94 (11.64) |
| OFC max tolerated dose mean (SD) | 4.42 (1.68) | | 8.00 (0.00) | 3.17 (1.17) | 4.68 (1.94) |
| PN-sIgE (kU/l) mean (SD) | 36.66 (40.04) | 0.00 (0.00) | 8.10 (11.06) | 31.97 (42.55) | 26.50 (36.90) |
| Max tolerated dose (g of protein): 1 = 0g, 2 = 0.003g, 3 = 0.012g, 4 = 0.043g, 5 = 0.137g, 6 = 0.447g, 7 = 1.378g, 8 (cumulative) = 4.481g | | | | | |

## Table S3: Selected clinical information for allergic patients separated by age and severity grades

| Cohorts | Children severity grade 1-2 (n=10) | Children severity grade 3-4 (n=12) | Adults severity grade 1-2 (n=5) | Adults severity grade 3-4 (n=37) | Total (n=64) |
|---|---|---|---|---|---|
| Male proportion | 0.40 | 0.50 | 0.20 | 0.38 | 0.39 |
| Age mean (SD) | 5.10 (1.66) | 8.42 (3.23) | 27.80 (4.49) | 27.22 (5.35) | 20.28 (10.77) |
| OFC max tolerated dose mean (SD) | 4.70 (1.57) | 4.58 (0.79) | 3.40 (1.14) | 3.81 (1.45) | 4.06 (1.39) |
| PN-sIgE (kU/l) mean (SD) | 34.53 (36.63) | 58.76 (44.33) | 48.62 (47.44) | 28.25 (36.21) | 36.54 (39.67) |
| Max tolerated dose (g of protein): 1 = 0g, 2 = 0.003g, 3 = 0.012g, 4 = 0.043g, 5 = 0.137g, 6 = 0.447g, 7 = 1.378g, 8 (cumulative) = 4.481g | | | | | |

## Table S4: Linear model of pS6 induction in cDCs after stimulation with aIgE

**Linear model pS6 in cDCs after stimulation with 10µg/ml anti-IgE**

pS6 ~ age (year) + activated basophils + PN-sIgE + severity score : age group

| Term | Estimate | Std.error | t.statistic | p value | Signif | $GVIF^{1/2*DF}$ |
|---|---|---|---|---|---|---|
| Intercept | 1.054 | 0.152 | 6.93 | 4.2e-09 | *** | |
| Age in years | -0.007 | 0.008 | -0.85 | 0.399 | | 1.59 |
| Activated basophils (decimal fraction) | 0.373 | 0.306 | 1.218 | 0.228 | | 1.12 |
| Severity (low vs high) in adults | 0.47 | 0.168 | 2.792 | 0.007 | ** | 1.3 |
| Severity (low vs high) in children | -0.055 | 0.175 | -0.316 | 0.753 | | 1.3 |
| **n** | | | **62** | | | |
| **adjusted $R^2$** | | | **0.188** | | | |
